# The impact of lockdown stress and loneliness during the COVID-19 pandemic on mental health among university students in Germany

**DOI:** 10.1038/s41598-021-02024-5

**Published:** 2021-11-22

**Authors:** Antonia M. Werner, Ana N. Tibubos, Lina M. Mülder, Jennifer L. Reichel, Markus Schäfer, Sebastian Heller, Daniel Pfirrmann, Dennis Edelmann, Pavel Dietz, Thomas Rigotti, Manfred E. Beutel

**Affiliations:** 1 Department of Psychosomatic Medicine and Psychotherapy, University Medical Center of the Johannes Gutenberg-University Mainz, Untere Zahlbacher Str. 8, 55131 Mainz, Germany; 2 Department of Work, Organizational and Business Psychology, Institute of Psychology, Johannes Gutenberg University Mainz, Mainz, Germany; 3 Institute of Occupational, Social and Environmental Medicine, University Medical Center of the University of Mainz, Mainz, Germany; 4 Department of Communication, Johannes Gutenberg University Mainz, Mainz, Germany; 5 Department of Sports Medicine, Rehabilitation and Disease Prevention, Institute of Sport Science, Johannes Gutenberg University Mainz, Mainz, Germany; 6 Leibniz Institute for Resilience Research, Mainz, Germany

**Keywords:** Disease prevention, Psychiatric disorders, Occupational health, Public health

## Abstract

The COVID-19 pandemic led to a shutdown of universities in Germany. In a longitudinal design, we compared mental health (depression, anxiety, somatic complaints) of university students in Germany before (June to August 2019) and in the course of the COVID-19 pandemic (June 2020) and determined the impact of pandemic-related stress and loneliness on students’ mental health in self-report online surveys. We investigated 443 participants (mean age 22.8 years), among them 77% female, and 10.4% medical students. A small increase of depression mean scores was observed (*F*(1,420) = 5.21; *p* = .023), anxiety and somatic complaints have not significantly changed. There was a medium increase in loneliness from pre-pandemic scores to the pandemic situation (*F*(1,423) = 30.56; *p* < .001). Analyzed with regression analyses, current loneliness and pre-pandemic distress represented the strongest associations with mental health during the pandemic. Additionally, health-related concerns during the pandemic were associated with symptoms of depression [*b* = 0.21; 95%CI(0.08; 0.34); *t* = 3.12; *p* = .002], anxiety [*b* = 0.07; 95%CI(0.01; 0.12); *t* = 2.50; *p* = .013], somatic complaints [*b* = 0.33; 95%CI(0.18; 0.47); *t* = 4.49; *p* < .001], and loneliness [*b* = 0.10; 95%CI(0.03; 0.17); *t* = 2.74; *p* = .006]. Social stress due to the pandemic situation was associated with loneliness [*b* = 0.38; 95%CI(0.32; 0.45); *t* = 11.75; *p* < .001]. The results imply that university students represent a risk group for psychosocial long-term ramifications of the pandemic.

## Introduction

Since the infectious disease COVID-19 has been declared a *pandemic* in March 2020, many measures in order to contain the disease such as maintaining physical distance have been installed by political administrations all over the world. In the beginning, the focus was on older persons who were at high risk for a more dangerous or even mortal course of the disease, and young adults were considered a low risk group for COVID-19 hospitalization and mortality^1^. In the course of the pandemic, the consequences on mental health have got increasing attention. Emerging evidence has pointed out a stress-related increase of levels of anxiety and depression, and of other symptoms of mental distress, underlining that the pandemic situation represents a stress event affecting people of all ages and different life situations^2^. In online surveys in the early phase of the pandemic, younger age^3–5^ and student status^6^ turned out as risk factors for heightened distress during the pandemic, along with female gender^6–8^.

Lockdown regulations (e.g. staying at home, physical distancing) may thwart important means for socializing, finding a partner, and building meaningful relationships which are of pivotal importance in early adulthood according to personality development theories^9, 10^. Thus, social isolation, reduced peer support, and loneliness may contribute to depressive symptoms in young adults during the pandemic^3, 11^. As the majority of mental disorders manifest in the second and third decade of life, and as there is a higher prevalence of mental disorders among college and university students than the general population^12, 13^, there is a need to look more closely at this group.

In the specific case of university students, the closing of universities during the COVID-19 pandemic lead to an abrupt loss of personal contacts with peers and faculty, postponement of curricula, research, practical work, and exchange programs^14^. The abrupt and often ill-prepared switch to online learning may additionally evoke stress^15^. Furthermore, the loss of temporary jobs (gastronomy, student, or teaching aides) could have compounded financial uncertainties^16^.

Recent studies indicated high levels of mental distress among university students at the beginning of the COVID-19 pandemic: in an online survey in the USA^4^, 48.1% of university students reported increased scores of depression, respectively 38.5% of anxiety. In a large online survey among Chinese college students (*N* = 89,588), 41.1% reported increased anxiety^17^. Similar figures were reported from university students from hot spot areas for COVID-19, like reports of students in New Jersey with a distress rate of almost 50%^7^ and a prevalence of PTSD of 16.3% among students in Wuhan^18^. Similar numbers were published in an online survey among French university students: 43% suffered from depression and 39% from anxiety^16^. Medical students in China perceived the health threat of COVID-19 as more serious, but also reported less anxiety and depression than non-medical students^19^. A systematic review mostly based on studies of Chinese university students, found a prevalence of anxiety of 28% among medical students who were assumed to be particularly burdened^20^. Among university students from Germany, the burden seems comparably high: for instance, Kohls et al. found a high proportion of students with elevated depression symptoms (37%) and compared the average levels of symptoms of depression between faculties with the humanities having the highest scores and students of medicine the lowest^21^. To date, studies on university students’ mental health during the COVID-19 pandemic have mostly relied on data obtained at one measurement point during the pandemic, realizing a cross-sectional design. As an exception, Li et al. (2021) studied a sample of university students twice, during the COVID-19 outbreak and its remission. While acute stress symptoms decreased, anxiety and depression increased^22^.

### Study objectives

As every educational system is different, and as countries were affected more or less by COVID-19, we were interested in the particular situation of students at a German university. Due to limitations in study design (mostly cross-sectional, without intra-individual development, or without a pre-pandemic assessment), and region of origin with few studies from German universities, there is little knowledge of how university students in Germany experienced the COVID-19 pandemic, and how it affected their mental health. Therefore, the current study investigated the following questions:

1. What is the symptom load of depression, anxiety, and somatic complaints (mental health) before and during the COVID-19 pandemic among university students in Germany?
2. What is the contribution of pandemic-related stress (health concerns, psychosocial stress situations, preventive behavior) as well as loneliness during the pandemic beyond pre-pandemic conditions (mental distress, gender, field of study) on university students’ mental health during the COVID-19 pandemic?

The study was performed by an ongoing student health initiative at the Johannes Gutenberg University (JGU), Mainz, Germany (“Healthy Campus Mainz”). The JGU is a full university covering a whole range of study programs (e.g. Social and Life Sciences, Humanities, Medicine, Teaching, Law and Economy, Science, Mathematics, and Informatics) with twelve faculties and around 32,000 students. The current study analyzed data from two online surveys, the first conducted before the pandemic (between June and August of 2019) and the second during the pandemic in June 2020.

We expected depression, anxiety, and somatic complaints to increase during the pandemic, particularly in female versus male students^8^ and in non-medical versus medical students^20^. Pandemic-related concerns, psychosocial stress, and loneliness were expected to contribute to mental distress during the pandemic after adjustment for pre-pandemic distress.

## Methods

### Data collection

Data were gathered in two online surveys conducted among the students of the Johannes Gutenberg University (JGU) Mainz, Germany. The first survey took place between June and August in 2019, the second in June 2020. Both surveys followed the same data collection procedure, with an e-mail invitation to all students through the central mailing list, and scheduled reminders. The questionnaires covered information about sociodemographic data (e.g. gender, age), mental health screeners, health behavior, and social support (see Reichel et al., for an overview of our methodology^23^). In the second survey, specific questions on COVID-19 have been added (pandemic-related concerns and psychosocial stress, adherence to preventive behavior in order to contain the pandemic). Recruitment procedures remained the same. Participation was voluntary and informed consent was obtained in advance to participation. Study approval was obtained by the ethical committees of the Medical Association of Rhineland-Palatinate (No. 2019-14336) and the Institute of Psychology of the JGU (No. 2020-JGU-psychEK-S008). Both studies were performed in accordance with the *Code of Ethics of the World Medical Association* (Declaration of Helsinki) for experiments involving humans and the *Ethical Principles and Guidelines for the Protection of Human Subjects of Research* by the American Psychological Association (APA).

### Study design

In 2019, an online survey was designed in order to investigate the university students’ health and to develop corresponding health promotion interventions. With the COVID-19 pandemic, another online survey based on the first one was created in March and April 2020, which was adapted to the pandemic situation and included additional questions on pandemic-related concerns, psychosocial stress, and preventive behavior. As we had planned from inception to monitor the students of our university in the course of the project, we installed a matching code in the first survey in order to have the possibility to identify participants in follow-up studies and to analyze intra-individual, longitudinal development. Thus, we integrated a participants’ code in the study conducted in 2019. Participants indicated the first two letters of their mother’s name, the first two letters of their mother’s birth name, the last letter of their first name, and the two figures of their birthday. The instruction to build the code was presented at the beginning of each survey. To identify students answering both surveys, the code was the variable we looked at first. Subsequently, we did plausibility checks on gender, excluding persons who changed their gender, as well as age, semester, and field of study.

Accordingly, in the current study, the individual scores of the main outcome variable mental health (depression, anxiety, somatic complaints), and also loneliness, self-perceived employability, and subjective general health of the two-time points of measurement (before and during the pandemic) were compared in a longitudinal study design, considering only those students who took the survey twice. Within this sample, different subgroups with regard to gender (male vs. female) and field of study (medical vs. non-medical) were analyzed as well. Additionally, the role of pre-pandemic and concurrent pandemic determinants of mental health was investigated.

### Measures

Regarding sociodemographic information, *age* was assessed in years. Participants indicated their *gender* as “male” = 1, “female” = 2, or “diverse” = 3 and their *field of study*. Answers were assigned as “non-medical” = 0 or “medical” = 1, including medicine and dentistry. In Mainz, students of medicine and dentistry are on a separate campus, the University Medical Center, apart from all other students. Due to their different location plus their particular involvement in the pandemic (medical students were asked to participate as volunteers in the University Medical Center), they might have experienced the pandemic substantially different than students of other subjects and were therefore chosen as a reference group in the analysis. Furthermore, participants were asked about their relationship status during the pandemic. Based on this, a binominal variable was built (“Single” = 0, “in a relationship” = 1).

The Patient Health Questionnaire 9 (PHQ-9)^24^ assessed nine symptoms (e.g. loss of interest, sleep disturbances, suicidal ideation) of Major Depression over the last 14 days on a scale between “not at all” = 0, “on single days” = 1, “on more than half of the days” = 2, and “almost every day” = 3. A sum score ≥ 10 indicates at least a moderate depressive disorder. The PHQ-9 has very good psychometric properties in the general population^25^. ‘The *Generalized Anxiety Disorder-2* (GAD-2)^26, 27^ assessed feelings of worry and nervousness based on the same answer options as in the PHQ-9. A sum score ≥ 3 indicates increased *generalized anxiety*. As an ultra-brief screening tool, the GAD-2 has acceptable psychometric properties and is a useful measure to screen for anxiety in the general population^26^. The Somatic Symptom Scale 8 (SSS-8)^28^ assessed eight somatic symptoms (e.g. back pain, headaches, dizziness) on five-point Likert-scales from “not at all” = 0, “a little bit” = 1, “somewhat” = 2, “quite a bit” = 3 to “very much” = 4. Scores ≥ 12 indicate a clinically relevant burden of *somatic complaints* (sum score between 0 and 32). *Loneliness* was measured by the German version of the three-item UCLA (University of California, Los Angeles) loneliness scale^29^: “how often do you… (1) …feel a lack of companionship?; (2) …feel isolated from others?; (3) …feel left out?”. Responses were scored on a five-point Likert scale from “never” = 0 to “very often” = 4 (sum scores 0 to 12). The scale has good psychometric properties in the general population^29–31^. Participants rated their *subjective health status* from “worst” = 0 to “best conceivable state of health” = 10. Positive outlook regarding the professional career after graduation was assessed as *self-perceived employability:* “How do you assess your future chances on the labor market?” (“very bad” = 0 to “very good” = 5).

In order to measure pandemic-specific experiences, we presented overall ten self-created items on the actual situation. Participants rated pandemic-related concerns about their own health, the health of a close person infected with the coronavirus, isolation/quarantine, supply shortages, and the economic impact of the pandemic on a seven-level scale from “no fear at all” = 1, “neutral” = 4, and “very strong fear” = 7. Additionally, they rated how much they feel stressed by not seeing family, friends, not pursuing hobbies, cancelled or postponed medical appointments, and if they needed psychological support on a scale from “not at all” = 1, “hardly” = 2, “somewhat” = 3, “quite” = 4, to “very” = 5. As we used self-developed items to assess pandemic-related experiences, we analyzed the psychometric properties of these overall ten items with a principal component analysis (PCA) in order to make the appropriate scoring. We conducted the PCA in a random sample within the cross-sectional sample of 2020 (*N* = 1247), independent from the longitudinal final sample. The first component was comprised of stress not meeting friends, not pursuing hobbies, not seeing important family members, fear of isolation/quarantine and concern for the economic consequences of the pandemic. We interpreted this score as the psychosocial stress during the pandemic situation (*social stress*). The second score consisted of concern for one’s health, concern for an infected person, fear of supply shortages, concern for cancelled and postponed medical appointments, and the wish for psychological support in overcoming the crisis. These aspects we summarized as *health issues* due to the pandemic.

In order to examine how participants were committed to recommended pandemic-related preventive behavior, they indicated, if they have followed a list of 10 behaviors during the last 7 days (e.g. “I have kept my distance to other people (at least 1.5 m).”). We counted the total number of behaviors endorsed to estimate *adherence* to pandemic containment measures, multiplied the number per 10, resulting in values between 0 and 100.

### Statistical analysis plan

Before conducting the main analyses in order to answer our two study questions, we describe the sample in terms of gender, age, and field of study. We also present descriptive data of the two cross-sectional samples in this regard.

For the first study question, we conducted six *Analyses of Variance* (ANOVA) with a mixed design, separately for each variable of interest (PHQ-9 score, GAD-2 score, SSS-8 score, loneliness score, subjective health, and self-perceived employability). The scores before and during the pandemic represented the within-subject factor *time*. As between-subject factors, *gender* and *field of study* were included in order to determine if male versus female and non-medical versus medical students were affected by the pandemic in similar ways. We report within-subject and between-subjects effects as well as the interaction effects of *time* × *gender*, and *time* × *field of study*. The effect size was estimated by partial eta^2^ (η_p_^2^); with η_p_^2^ ≥ 0.01 indicating a small, η_p_^2^ ≥ 0.06 a medium, and η_p_^2^ ≥ 0.14 a large effect.

In order to answer the second study question, multiple regression analyses identified determinants of mental health (depression, anxiety, somatic complaints) during the pandemic. In order to do so, we considered the role of pandemic-specific variables to make sure that possible changes did nor only increase over time which would have been observable also without the pandemic. We used the pandemic-related *social stress*, the pandemic-related *health issues*, and *adherence* to pandemic containment measures as pandemic-specific variables in all our analyses.

In Model 1, we took into account pre-pandemic characteristics (pre-pandemic levels of mental health, gender, field of study), and we added as further variables relationship status (during the pandemic), loneliness during the pandemic, and our newly developed pandemic variables (Model 2).

In order to understand the specific role of loneliness during the pandemic, another multiple regression analysis was conducted for loneliness during the pandemic as an outcome with pre-pandemic loneliness, gender, and field of study as predictors in Model 1. In a second step, we considered additionally relationship status during the pandemic, and the specific pandemic variables (Model 2). All statistical analyses were performed with IBM SPSS, version 26.

### Additional analyses

We describe relative frequencies separately for each of the ten items of the two pandemic-related variables *social stress* and *health issues* by building categorical binominal variables. Answers between 1 and 4 were assigned to “no agreement” = 0, and answers between 5 and 7 “agreement” = 1 or answer options 1 and 2 indicating “no or little” = 0 and answers from 3 to 5 “at least moderate” perceived stress/wish for psychological support = 1, respectively, in accordance with the Likert-Scale of the item. Furthermore, we describe how frequently participants indicated that they followed a certain pandemic containment measure. We do so for the total sample as well as separately for gender (male vs. female), and field of study (medical vs. non-medical), respectively.

## Results

A total number of 443 university students answered both surveys, out of 4,351 students who took part in the first and 3,066 students from the second survey corresponding to 10 or 14%, respectively (Table 1). Their mean age was 22.8 years at baseline (*SD* = 3.3 years); 77% were female, and 10.4% were medical students. During the pandemic, 53% reported to be in a relationship. Due to the small group size, participants who indicated themselves as diverse (*n* = 2) were excluded from the main analyses. For our focus on intra-individual change, we calculated post hoc power analyses for all the conducted statistical procedures and the according sample sizes. With regard to the ANOVAs, there was an excellent power of more than 99% to detect small effects. Looking at determinants of mental health among our participants with multiple regression analyses, the power was also excellent with more than 99% for *R*^2^, change in *R*^2^, and single predictors. Our longitudinal subsample is comparable with the cross-sectional samples in terms of the gender distribution and with regard to the proportions of participants with different fields of study (see Table 1). All samples had a higher proportion of female participants compared to the proportion of female students at the university^23^. In advance of our analyses we checked for normality of our data. We assumed normality of the data, as mean and median were comparable in all variables. Furthermore, due to the sufficient sample size, we concluded to have a data base robust enough against a possible violation of normality.

**Table 1:** Frequencies of gender and field of study in the study sample before and during the pandemic.

	Analyzed longitudinal sample (*N* = 443)	Survey 2019 (*N* = 4351)	Survey 2020 (*N* = 3066)
**Gender**			
Women, No. (%)	341 (77.0)	3065 (70.4)	2225 (72.6)
Men, No. (%)	100 (22.6)	1246 (28.6)	821 (26.8)
Diverse, No. (%)	2 (0.5)	39 (0.9)	20 (0.7)
**Field of study**			
STEM^a^, No. (%)	71 (16.0)	783 (18.0)	506 (16.5)
Social sciences, No. (%)	94 (21.2)	774 (17.8)	493 (16.1)
Humanities, No. (%)	87 (19.6)	871 (20.1)	630 (20.5)
Medicine, No. (%)	46 (10.4)	582 (13.4)	341 (11.1)
Law and economics, No. (%)	49 (11.1)	576 (13.3)	479 (15.6)
Teaching, No. (%)	92 (20.8)	665 (15.3)	510 (16.6)
Other, No. (%)	3 (0.7)	91 (2.1)	53 (1.7)
**Relationship status (during the pandemic)**			
Single, No. (%)	193 (43.6)	–	1349 (44.0)
In a relationship, No. (%)	235 (53.0)	–	1520 (49.6)

^a^Science, Technology, Engineering, and Mathematics.

### Change in mental health before and during the COVID-19 pandemic

Table 2 presents the mean scores and standard deviations for symptoms of depression and anxiety, somatic complaints, loneliness, self-perceived employability, and subjective general health in the analysis sample with regard to the within-subject factor *time of the survey* (before vs. during the pandemic), and the two between-subject factors *gender* (male vs. female) and *field of study* (non-medical vs. medical). In case of missing data in one of the relevant variables in a model, we applied list-wise deletion of participants, resulting in slightly different sample sizes for the different ANOVAs.

**Table 2:** Means, standard deviations, and analyses of variance in depression, general anxiety, somatic symptoms, loneliness, self-perceived employability, and subjective health by time of survey (pre-pandemic vs. during the pandemic), gender (male vs. female), and field of study (medical vs. other).

	Before the pandemic					During the pandemic					Time (within-subject factor)			Gender (between-subject factor)			Field of study (between-subject factor)		
	All	Gender		Field of study		All	Gender		Field of study										
		Male	Female	Med	No med		Male	Female	Med	No med									
	*M* (SD)	*M* (SD)	*M* (SD)	*M* (SD)	*M* (SD)	*M* (SD)	*M* (SD)	*M* (SD)	*M* (SD)	*M* (SD)	*F* (*df*)	*p*	η_p_^2^	*F* (*df*)	*p*	η_p_^2^	*F* (*df*)	*p*	η_p_^2^
Depression (PHQ-9), *N* = 424	7.49 (4.87)	6.91 (4.76)	7.66 (4.90)	6.62 (5.33)	7.60 (4.82)	8.49 (5.31)	7.82 (5.39)	8.69 (5.27)	7.20 (5.57)	8.64 (5.26)	5.21 (1, 420)	**.023**	.012	4.51 (1, 420)	**.034**	.011	5.11 (1, 420)	**.024**	.012
Anxiety (GAD-2), *N* = 425	2.06 (1.57)	1.60 (1.53)	2.20 (1.56)	1.82 (1.51)	2.09 (1.57)	2.12 (1.73)	1.78 (1.75)	2.22 (1.72)	1.76 (1.54)	2.16 (1.75)	1.88 (1, 421)	.171	.004	5.92 (1, 421)	**.015**	.014	2.80 (1, 421)	.095	.007
Somatic complaints (SSS-8), *N* = 425	8.92 (4.89)	6.89 (4.35)	9.52 (4.89)	7.78 (4.97)	9.06 (4.87)	9.48 (5.52)	6.96 (5.03)	10.22 (5.44)	8.04 (5.27)	9.65 (5.53)	0.29 (1, 421)	.592	.001	13.73 (1, 421)	**˂.001**	.032	3.97 (1, 421)	**.047**	.009
Subjective Health, *N* = 428	7.53 (1.59)	7.59 (1.46)	7.52 (1.63)	7.56 (1.88)	7.53 (1.63)	7.40 (1.66)	7.53 (1.66)	7.36 (1.66)	7.36 (1.90)	7.40 (1.63)	0.23 (1, 424)	.630	.001	4.17 (1, 424)	**.042**	.010	1.30 (1, 424)	.255	.003
Loneliness, *N* = 427	3.89 (2.73)	4.08 (2.89)	3.83 (2.68)	3.51 (2.64)	3.93 (2.74)	5.89 (2.69)	5.49 (2.85)	6.01 (2.63)	5.18 (2.20)	5.98 (2.73)	30.56 (1, 423)	**˂.001**	.067	1.95 (1, 423)	.163	.005	4.75 (1, 423)	**.030**	.011
Self-perceived employability *N* = 398	4.90 (1.29)	5.17 (1.36)	4.82 (1.26)	6.33 (0.78)	4.73 (1.23)	4.82 (1.41)	4.95 (1.55)	4.79 (1.37)	6.26 (1.36)	4.65 (1.31)	0.64 (1, 394)	.424	.002	4.99 (1, 394)	.**013**	> .001	64.45 (1, 394)	**˂.001**	.141

*Med* Medical students, *No med* Non-medical students, *PHQ-9* Patient health questionnaire-9, *GAD-2* Generalized anxiety disorder-2, *SSS-8* Somatic symptoms scale-8. Different *N* due to list-wise deletion.

There was a significant increase in symptoms of depression (*n* = 424; small effect size), while no significant increase was found for both anxiety (*n* = 425) and somatic complaints (*n* = 425). Loneliness increased over time (*n* = 427; medium effect size). No significant differences were found for subjective general health (*n* = 428) and self-perceived employability (*n* = 398; Table 2).

Looking at between-subject effects, female students reported on average higher symptom load of depression, anxiety, somatic complaints, and lower subjective general health and self-perceived employability compared to male students (Table 2). There were no significant gender differences in loneliness.

Regarding the field of study, non-medical students reported more symptoms of depression, loneliness, and somatic complaints than medical students. The strongest difference became evident regarding self-perceived employability, which medical students described as higher than non-medical students. There were no differences with regard to anxiety, and subjective general health.

**Figure 1:**
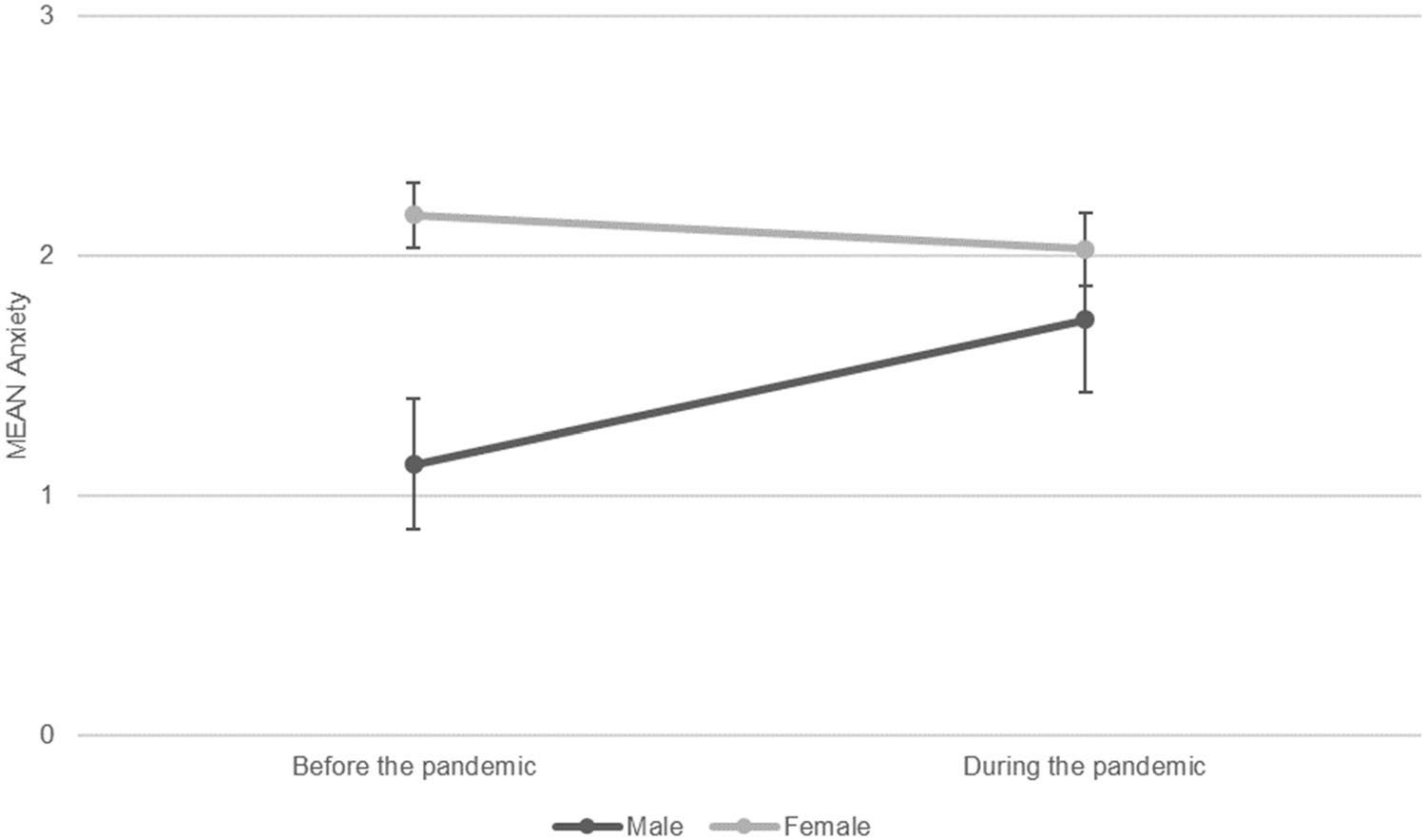
Anxiety predicted by time (before vs. during pandemic) and gender (male vs. female). *Note F*(1,421) = 5.03, *p* = .025, small effect size.

There were no significant interaction effects of *time* × *gender* or *time* × *field of study* besides one interaction between *time* × *gender*, and anxiety as dependent variable: male students reported an increase of anxiety symptoms during the pandemic, while female students, reported rather constant levels, resulting in an approximation of anxiety scores in the female and male students (*F*(1,421) = 5.03, *p* = 0.025, small effect, Fig. 1). Second-order interactions (*time* × *gender* × *field of study*) were not analyzed due to the small group size.

### Determinants of mental health during the COVID-19 pandemic

As indicated by Spearman Rank correlations between the analyzed variables, both pandemic-related variables (*social stress* and *health issues*) were associated with mental distress, loneliness, somatic complaints, and gender (Table 3).

**Table 3:** Spearman Rank correlations between pandemic-related variables and outcome measures, gender, age, and relationship status during the pandemic.

	Health issues (pandemic)	Adherence (pandemic measures)	SSS-8 (pandemic)	PHQ-9 (pandemic)	Loneliness (pandemic)	GAD-2 (pandemic)	Gender	Age	Relationship status (pandemic)
Social stress (pandemic)	.38**	.10*	.33**	.32**	.49**	.24**	.22**	.002	< .01
Health issues (pandemic)		.20**	.41**	.40**	.36**	.36**	.14**	.10*	− .07
Adherence (pandemic measures)			.14**	.09	.04	.07	− .01	− .01	.07

Bold values are statistically significant.

*SSS-8* Somatic symptoms scale-8, *PHQ-9* Patient health questionnaire-9, *GAD-2* Generalized anxiety disorder-2.

*p < .05 (two-tailed); **p < .01 (two-tailed).

Table 4 shows the multiple linear regression models for the main outcome of mental health during the pandemic (depression, anxiety, and somatic complaints). Depression (*n* = 418) during the pandemic was predicted most strongly by pre-pandemic depression (Model 1, *R*^2^ = 0.39, *p* < 0.001). Including loneliness (during the pandemic) and pandemic-related variables, Model 2 explained 51% of variance (*R*^2^ = 0.51, *p* < 0.001) with loneliness, and pandemic-related health issues as additional significant predictors.

**Table 4:** Linear regressions of associations between depression, anxiety, and somatic symptoms with gender, field of study, relationship status (model 1), and loneliness, social stress due to the pandemic, health issues due to the pandemic, and adherence to pandemic containment measures (model 2).

	Depression (PHQ-9)						General anxiety (GAD-2)						Somatic symptoms (SSS-8)					
	Model 1 (*R*^2^ = .39***)						Model 1 (*R*^2^ = .22***)						Model 1 (*R*^2^ = .42***)					
	*B*	*SE*	95% CI L	95% CI U	*t*	*p*	*B*	*SE*	95% CI L	95% CI U	*T*	*p*	*B*	*SE*	95% CI L	95% CI U	*t*	*p*
Pre-pandemic distress^a^	0.66	0.04	0.58	0.75	16.03	< **.001**	0.51	0.05	0.42	0.61	10.55	> **.001**	0.69	0.04	0.61	0.78	16.05	> **.001**
Gender	0.39	0.47	− 0.54	1.32	0.83	.409	0.15	0.18	− 0.20	0.49	0.83	.405	1.34	0.49	0.38	2.31	2.73	**.007**
Field of study	− 1.05	0.67	− 2.36	0.26	− 1.58	.116	− 0.33	0.25	− 0.82	0.15	− 1.35	.178	− 0.90	0.68	− 2.24	0.44	− 1.33	.186

	Depression (PHQ-9)						General anxiety (GAD-2)						Somatic symptoms (SSS-8)					
	Model 2 (*R*^2^ = .51***)						Model 2 (*R*^2^ = .30***)						Model 2 (*R*^2^ = .50***)					
	*B*	*SE*	95% CI L	95% CI U	*t*	*p*	*B*	*SE*	95% CI L	95% CI U	*t*	*p*	*B*	*SE*	95% CI L	95% CI U	*t*	*p*
Pre-pandemic distress^a^	0.54	0.04	0.46	0.62	13.75	< **.001**	0.42	0.05	0.33	0.52	8.78	< **.001**	0.59	0.04	0.51	0.68	13.85	< **.001**
Gender	− 0.13	0.44	− 0.99	0.72	− 0.31	.759	0.04	0.17	− 0.30	0.38	0.25	.802	0.95	0.47	0.02	1.88	2.01	**.045**
Field of study	− 0.67	0.60	− 1.86	0.51	− 1.12	.263	− 0.24	0.24	− 0.71	0.22	− 1.02	.306	− 0.80	0.64	− 2.06	0.47	− 1.24	.215
Relationship status	− 0.14	0.38	− 0.89	0.60	− 0.38	.702	0.12	0.15	− 0.18	0.41	0.78	.435	0.10	0.40	− 0.69	0.89	0.25	.805
Loneliness	0.59	0.08	0.43	0.75	7.08	< **.001**	0.17	0.03	0.11	0.24	5.34	< **.001**	0.27	0.09	0.10	0.44	3.08	**.002**
Social stress	0.04	0.07	− 0.09	0.18	0.67	.504	− 0.2	0.03	− 0.07	0.03	− 0.81	.418	0.11	0.07	− 0.03	0.25	1.54	.124
Health issues	0.21	0.07	0.08	0.34	3.12	**.002**	0.07	0.03	0.01	0.12	2.50	**.013**	0.33	0.07	0.18	0.47	4.49	** < .001**
Adherence	− 0.01	0.01	− 0.04	0.02	− 0.52	.604	0.01	0.01	− 0.01	0.01	− 0.35	.726	− 0.01	0.01	− 0.04	0.02	− 0.64	.524

Bold values are statistically significant.

*CI* Confidence interval, *PHQ-9* Patient health questionnaire-9, *GAD-2* Generalized anxiety disorder-2, *SSS-8* Somatic symptoms scale-8.

^a^In the corresponding outcome variable (PHQ-9, GAD-2, SSS-8, respectively).

Variance of anxiety (*n* = 419) was explained in Model 1 with 22% (*R*^2^ = 0.22, *p* < 0.001), and the significant predictor pre-pandemic anxiety. In Model 2, 30% of variance in anxiety levels was explained (*R*^2^ = 0.30, *p* < 0.001), with pre-pandemic anxiety, loneliness, and pandemic-related health issues as significant predictors.

Somatic symptoms (*n* = 419) were associated with pre-pandemic somatic symptom load and additionally with gender (Model 1, *R*^2^ = 0.42, *p* < 0.001). In Model 2, additionally loneliness and pandemic-related concerns about health (*health issues*) contributed significantly to the explained variance of somatic symptoms (Model 2, *R*^2^ = 0.50, *p* < 0.001).

With regard to loneliness during the pandemic (*n* = 419), pre-pandemic loneliness and female gender were relevant predictors in Model 1 with 17% explained variance. In Model 2, it became evident that additionally to pre-pandemic loneliness, not having a relationship during the pandemic, higher psychosocial stress (*social stress*), and more pandemic-related health concerns (*health issues*), explained 44% of variance in pandemic loneliness scores (Table 5).

**Table 5:** Linear regressions of associations between loneliness and gender, field of study, relationship status (model 1), and social stress due to the pandemic, health issues due to the pandemic, and adherence to pandemic containment measures (model 2).

Outcome: loneliness	*B*	*SE*	95% CI L	95% CI U	*t*	*p*
**Model 1 (*R***^**2**^ = .17***)						
Pre-pandemic loneliness	0.93	0.04	0.31	0.48	8.84	< **.001**
Gender	0.69	0.28	0.14	1.24	2.46	**.014**
Field of study	− 0.64	0.40	− 1.42	0.15	− 1.60	.110
**Model 2 (*R***^**2**^ = .44***)						
Pre-pandemic loneliness	0.35	0.04	0.27	0.42	9.12	< **.001**
Gender	0.07	0.24	− 0.40	0.54	0.29	.776
Field of study	− 0.51	0.33	− 1.16	0.14	− 1.54	.124
Relationship status	− 0.54	0.21	− 0.95	− 0.14	− 2.64	**.009**
Social stress	0.38	0.03	0.32	0.45	11.75	< **.001**
Health issues	0.10	0.04	0.03	0.17	2.74	**.006**
Adherence	− 0.01	0.01	− 0.03	< − 0.01	− 1.68	.093

Bold values are statistically significant

### Additional analyses

**Figure 2:**
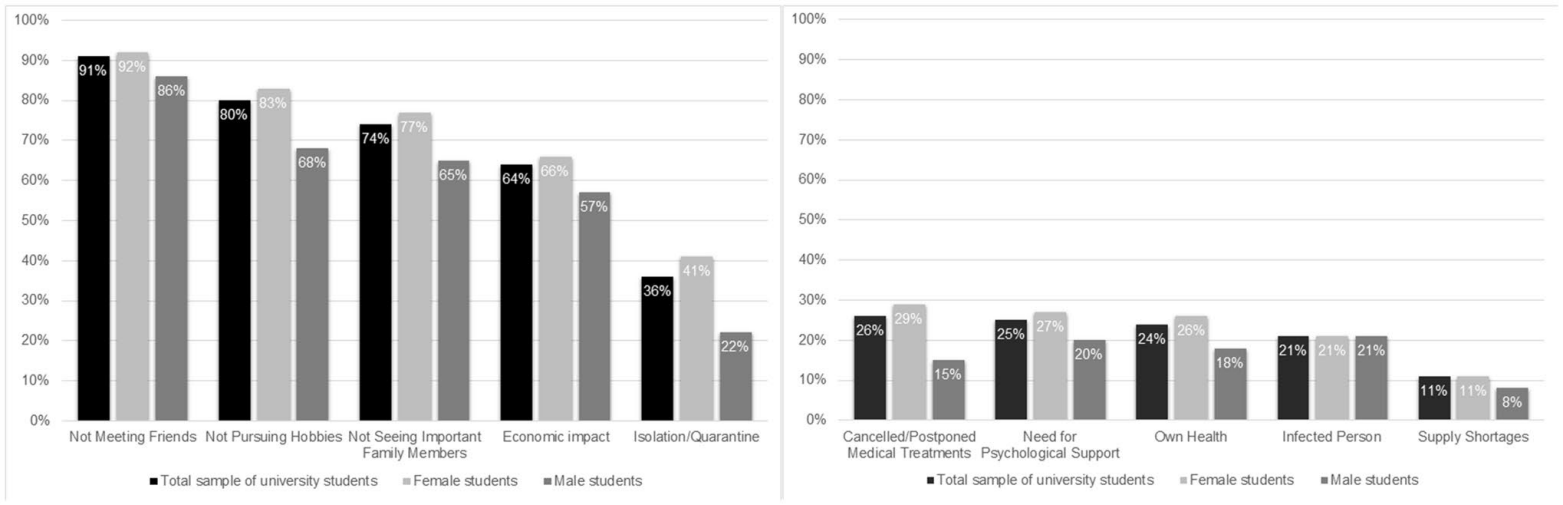
**(a)** Social stress due to the COVID-19 pandemic reported by university students in June 2020 (N = 443). (**b**) Health issues due to the COVID-19 pandemic reported by university students in June 2020 (N = 443). *Note*. Participants answered five questions on pandemic-related items. Answers between 1 and 4 were assigned to “no agreement” = 0, and answers between 5 and 7 “agreement” = 1 on the 7-point Likert-Scale; answer options 1 and 2 indicated “no or little” = 0 and answers from 3 to 5 “at least moderate” perceived stress/wish for psychological support = 1 on the 5-point Likert-Scale, respectively. Scoring was in accordance with the answer options of the item.

With regard to *social stress* due to the pandemic, the great majority felt stressed by no longer meeting friends (91%) or family members (74%) and not pursuing hobbies (80%) and almost two-thirds articulated concerns over the economic impact (64%). About one-third was concerned about isolation or quarantine (36%). Compared to male students, female students were significantly more stressed by not seeing important family members (65% vs. 77%, χ^2^ = 5.27, *p* = 0.022) and not pursuing interests/hobbies (68% vs. 83%, χ^2^ = 11.21, *p* = 0.001). They were by far more concerned for isolation/quarantine than male students (41% vs. 22%, χ^2^ = 11.46, *p* = 0.001; see Fig. 2a). There were no differences with regard to field of study, contrasting medical versus non-medical students.

In terms of pandemic-related concerns about health (*health issues*), 26% had to postpone medical treatments, and 25% desired psychological support in overcoming the crisis (Fig. 2b). There was a concern as well about one’s own or the health of an infected person (24%, resp. 21%). Fear for the shortage of supply was comparably rare (11%). Female students were more concerned than male students by cancelled medical treatments (15% vs. 29%, χ^2^ = 7.94, *p* = 0.005). There were no further gender differences. No differences between medical and non-medical students with regard to pandemic-related health concerns were observed.

**Figure 3:**
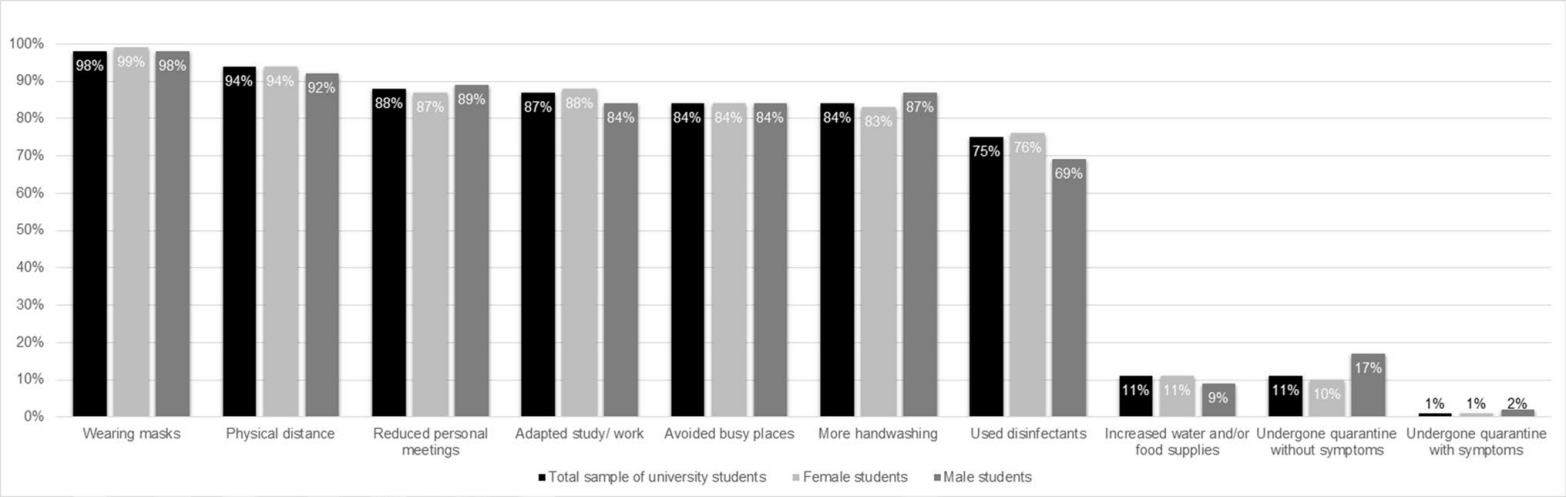
Adherence to pandemic containment measures during the COVID-19 pandemic reported by university students in June 2020 (N = 443).

Almost all participants (98%) reported that they have worn masks and maintained physical distance (94%) over the last seven days. Personal meetings had been reduced by 88% of the sample. They adapted their study or work (87%), avoided busy places (84%), and practiced more hand-washing (84%). Male students reported more often than female students to have put themselves into quarantine even though they had no symptoms (17.0% vs. 10%, χ^2^ = 4.36, *p* = 0.037). There were no other gender differences (Fig. 3). Medical students did not differ from non-medical students regarding the reported preventive behaviors.

Looking at the overall scores, participants followed on average 6.32 preventive behaviors (*SD* = 1.31). There was no gender difference regarding pandemic-related preventive behavior (*M*_*femal*e_ = 6.32 vs. *M*_*male*_ = 6.31; *t* = 0.09, *df* = 435, *p* = 0.928). There were no differences between medical and non-medical students regarding these overall scores in the three pandemic-related measures.

## Discussion

In the context of a university-based health promotion program, we investigated, first, mental health before and in the early course of the COVID-19 pandemic, and second, the role of pandemic-related stress, and preventive behavior. In line with the hypotheses, the mean scores of depression increased during the pandemic intra-individually compared to pre-pandemic scores in our sample. Yet, contrary to the hypotheses, no significant increase was found for anxiety and somatic complaints, respectively. Likewise, no significant change neither was observed in self-perceived employability nor subjective general health. From our point of view, the results should be interpreted in the context of the time period when the surveys took place. In June 2020, the first wave of COVID-19 was receding and during the summer months, there was no harsh lockdown in Germany. Therefore, students could have felt less stressed by feelings of anxiety or somatic complaints during the early phase of the pandemic and were more affected by the social restrictions. This could explain our result, that loneliness increased the most from pre-pandemic scores to pandemic scores with a medium effect size.

Female versus male and also non-medical versus medical students reported more mental health issues (depression, anxiety, somatic complaints), consistent with previous findings regarding gender^6–8^ and field of study^20^. Previous findings comparing medical and non-medical students regarding depression have been inconclusive^32^, and it must be considered that medical students may become socialized to disclaim mental health problems during their training^33^. On the other side, there was only one interaction effect, which was between time and gender for anxiety: while male students’ anxiety scores increased during the pandemic, female students’ scores remained at the same level (small effect). Altogether, contrary to previous studies^8, 20^ and to our hypotheses, the absence of interactions indicated that neither the mental distress of female nor non-medical students increased more than male or medical students’ distress. The interaction between anxiety and gender even indicated the opposite effect, namely that male students’ anxiety increased more than female students’ anxiety during the pandemic.

The second study question focused on pandemic-related stress and the adherence to preventive behavior during the pandemic. The regression analyses demonstrated that pre-pandemic mental health scores were the strongest predictors for depression, anxiety, and somatic complaints, respectively. Loneliness during the pandemic represented the second strongest association with mental health issues. Gender was only associated with somatic symptoms, with female students reporting more somatic complaints. Furthermore, the variable health issues due to the pandemic was a significant predictor in all regression analyses: the more participants had concerns about their health, were worried about an infected person, postponed medical treatments and stressed by possible isolation/quarantine or in need for psychological support, the more they reported mental distress and somatic symptoms. Higher levels of pandemic-related social stress on the other hand played no role for pandemic depression and anxiety scores or somatic complaints. It was not directly associated with these outcomes. Likewise, adherence to pandemic containment measures was not a significant predictor in any of the regression models.

Interestingly, the outcome loneliness during the pandemic was only predicted to a comparably small degree by pre-pandemic loneliness. Unlike depression, anxiety, and somatic complaints, the degree of reported loneliness was associated with pandemic factors much more, with an increasing explained variance from 16 to 43%. Therefore, it can be concluded that pandemic-related stress might have also contributed indirectly through loneliness to depression, anxiety, and somatic complaints during the pandemic.

The observation that following a higher number of preventive behavior recommendations was not associated with any of the distress outcomes could indicate that taking social responsibility, feeling part of the society and making a social contribution in order to fight the pandemic may be a way to cope adaptively with the pandemic situation. Furthermore, those persons who followed the pandemic rules may have found creative ways to keep at least some personal contact for example by balancing potentially risky contacts by self-chosen quarantine and utilizing digital contact possibilities^34^.

The prevailing public health strategy has aimed at containing the spread of COVID-19 by implementing physical distancing (along with hygiene measures such as wearing facial masks), avoiding close social contacts beyond the immediate household. Indeed, almost all of the participating university students reported following rules such as not meeting friends. Therefore, it is not surprising that loneliness has been identified as the strongest pandemic-related factor promoting mental health issues of depression, anxiety and somatic symptoms. Researchers on loneliness already expected that loneliness and social isolation are of high relevance for mental health issues during the pandemic^35^. Notably in this regard: gender differences were observed regarding concerns about quarantine, which affected female almost twice as often as male students. Likewise, they suffered more by not meeting family, not pursuing hobbies, and by cancelled medical treatments than male students. Both articulated comparably often the need for psychological support. These results could reflect that female students might have felt more stressed by some of the social and health-related aspects due to the pandemic than male students. Overall, we found that most of the students followed the recommended containment measures, even if the pandemic situation was accompanied by more loneliness and more depressive symptoms.

Unlike most published studies on university students’ mental health, which are based on cross-sectional data, we analyzed the same participants at two measurement points, conducted at the same time of each year, with the same recruitment strategies. By a longitudinal design, we could investigate intra- and inter-individual changes of mental health from pre-pandemic conditions to the situation during the pandemic among university students. The correlational patterns of our pandemic-specific questions look plausible and support the validity of the measures.

Yet, as the investigation was conducted only at one German university, and due to a low response rate, generalization of the findings is limited. Regarding our study design, selection bias might be present, preferentially reaching those who were strongly preoccupied by health topics in general, and the pandemic in particular. Additionally, comparably more female than male students took part in the study, as female students seem to be more willing to participate in such surveys than male students^36^. While the overall response rate was comparable to other university student surveys with about 10% (2020) to 14% (2019)^4^, there was a further reduction in sample size by analyzing only those participating at both measurement points. Yet, the longitudinal sample was comparable to the two cross-sectional samples in terms of gender and field of study.

The increase of depression, anxiety, and somatic complaints was smaller than expected from cross-sectional observations. Loneliness was the most substantial consequence of the pandemic. Given their high levels of distress before and during the pandemic, and their pandemic-related health concerns and social stress, however, university students represent an under-estimated risk group for the social and economic ramifications of the pandemic. This is underscored by one in four participants indicating a need for psychological support.

Future research should focus on the further development of mental health promotion among university students in the course of the COVID-19 pandemic, as our investigation took part comparably early after the first general lockdown in Germany, and during the first semester of a university, which was largely closed for attendance.

Concerns and psychosocial stress of university students during the pandemic (and post-pandemic) must be specifically identified (e.g. academic, social, mental health, financial), in order to offer the appropriate support. Given the limitations of still ongoing COVID-19 containment measures, and a presumable affinity for internet use, coordinated online support could be a suitable way to reduce loneliness among university students and their risk of mental disorders^37^.

## Data Availability

The dataset generated during and/or analyzed during the current study is available in the Open Science Framework (OSF) repository, doi: 10.17605/OSF.IO/Z7QSY.
